# 多孔氮化硼掺杂聚吡咯-2,3,3-三甲基吲哚固相微萃取涂层的制备及多环芳烃的检测

**DOI:** 10.3724/SP.J.1123.2023.03015

**Published:** 2023-09-08

**Authors:** Jie DU, Pengchao SUN, Menglu ZHANG, Zete LIAN, Fenggang YUAN, Gang WANG

**Affiliations:** 1.中国石油新疆油田分公司实验检测研究院, 新疆 克拉玛依 834000; 1. Experimental Testing Research Institute of PetroChina Xinjiang Oilfield Company, Karamay 834000, China; 2.中国石油天然气集团公司砾岩油气藏勘探开发重点实验室, 新疆 克拉玛依 834000; 2. China National Petroleum Corporation Key Laboratory for Exploration and Development of Conglomerate Oil and Gas Reservoirs, Karamay 834000, China; 3.中国石油新疆油田分公司采油一厂, 新疆 克拉玛依 834000; 3. No.1 Petrol Factory of PetroChina Xinjiang Oilfield Company, Karamay 834000, China; 4.中国石油新疆油田分公司采气一厂, 新疆 克拉玛依 834000; 4. No.1 Gas Factory of PetroChina Xinjiang Oilfield Company, Karamay 834000, China

**Keywords:** 固相微萃取, 气相色谱, 循环伏安法, 多环芳烃, 环境水样, solid-phase microextraction (SPME), gas chromatography (GC), cyclic voltammetry (CV), polycyclic aromatic hydrocarbons (PAHs), environmental water samples

## Abstract

多环芳烃(PAHs)是持久性有机污染物中的一种,大部分具有较强的致癌、致畸和致突变性,对生态环境和人类健康易造成严重威胁。由于环境样品基质复杂且其中PAHs含量低,因此在仪器分析之前需要对环境样品进行必要的前处理。萃取材料的特性是决定大部分前处理技术萃取效率的关键。基于此,本文以低成本且富含较多官能团的吡咯(py)、2,3,3-三甲基吲哚(2,3,3-TMe@In)为单体,多孔氮化硼为掺杂物,采用电化学循环伏安法制备出多孔氮化硼掺杂聚吡咯-2,3,3-三甲基吲哚(Ppy/P2,3,3-TMe@In/BN)复合涂层,通过扫描电子显微镜、热稳定性分析、傅里叶红外光谱等手段对Ppy/P2,3,3-TMe@In/BN进行表征,结果表明:该涂层呈现出多孔、多褶皱的枝状结构,该结构有利于增加涂层的比表面积,从而实现对PAHs的大量富集;在320 ℃解吸温度下,涂层材料的色谱基线基本稳定,表明该涂层具有良好的热稳定性。将其修饰在不锈钢丝表面制成固相微萃取涂层,结合气相色谱-氢火焰离子化检测器,对影响萃取和分离萘(NAP)、苊(ANY)、芴(FLU)3种PAHs的条件进行优化,建立了用于以上3种PAHs检测的分析方法。该方法具有检出限低(10.6~14.5 ng/L, *S/N*=3)、稳定性好、萃取效率高等优势。将该方法应用于2种环境水样中3种PAHs的检测,在水样1中检测到少量的ANY(1.39 μg/L)。通过向2种水样中加入低(1 μg/L)、中(10 μg/L)、高(50 μg/L)3个水平的标准溶液考察了该方法的可靠性,得到了满意的回收率(82.5%~113.9%)。实验结果表明,所建立的分析方法可实现对环境水样中这3种PAHs的有效检测。

## Abstract (en)

Most polycyclic aromatic hydrocarbons (PAHs), which are persistent organic pollutants, have strong carcinogenicity, teratogenicity, and mutagenicity, and pose serious threats to the ecological environment and human health. Owing to the complexity of the matrix and low PAH content of environmental samples, separating and enriching PAHs in environmental samples is necessary prior to their detection. Solid-phase microextraction (SPME) technology is commonly used to detect PAHs owing to its advantages of simple operation, online connection with other instruments, low solvent usage, and integrability of sampling separation, enrichment, and desorption. The extraction coating is the core of this technology, and the type and thickness of the coating are important factors affecting the sensitivity and accuracy of the analysis. Common commercial extraction coatings include polydimethylsiloxane and quartz fiber; however, these materials have a number of disadvantages, such as poor thermal stability and high cost. Several methods, including electrochemical, sol-gel, molecular imprinting, and other coating methods, have been developed to prepare SPME coatings. Electrochemical methods have attracted considerable attention because of their simplicity, short duration, and high coating stability. In the development of an electrochemical method, the selection of the conductive polymer is of particular importance. Polypyrroles (Ppy) are easily synthesized and have numerous advantages, such as good conductivity and stable chemical properties. Thus, their use as a substrate material for SPME coatings is beneficial for improving the overall stability of the coating. Copolymerization with other polymers can enhance the adsorption performance of such coatings via synergistic effects. When doped with inorganic materials with high thermal stability, the composite coating can exhibit high temperature resistance.

In this study, a porous boron nitride-doped Ppy-2,3,3-trimethylindole (Ppy/P2,3,3-TMe@In/BN) composite was prepared as a new SPME copolymer coating to detect three PAHs: naphthalene (NAP), acenaphthene (ANY), and fluorene (FLU). Scanning electron microscopy, thermal stability analysis, Fourier transform infrared spectroscopy, and other techniques were used to characterize the Ppy/P2,3,3-TMe@In/BN composite coating. The results showed that the coating featured a large number of porous and wrinkled dendritic structures, which increased the specific surface area of the composite coating and enabled the extensive enrichment of the three PAHs. When the sample inlet temperature of the chromatograph is 320 ℃, the chromatographic baseline of the coating is basically stable. Compared with commercial coatings, the prepared coating had better thermal stability. The coating formed stable intermolecular forces with the three PAHs owing to its numerous carbon-carbon double bonds (C=C), hydrogen bonds, and other structures, thereby achieving excellent enrichment of the target analytes. Compared with Ppy, Ppy/PIn, Ppy/P2,3,3-TMe@In, Ppy/BN, and polydimethylsiloxane (PDMS) coatings, the prepared Ppy/P2,3,3-TMe@In/BN composite coating exhibited better extraction effects for the three PAHs. The Ppy/P2,3,3-TMe@In/BN composite coating was polymerized on the surface of a stainless-steel wire by cyclic voltammetry and combined with gas chromatography-hydrogen flame ionization detection (GC-FID) to optimize the conditions influencing the extraction and separation of the three PAHs, thereby establishing a highly sensitive analytical method for detecting NAP, ANY, and FLU. This method had low limits of detection (LODs) of 10.6-14.5 ng/L (*S/N*=3) and high stability. The SPME-GC-FID method was used to detect the three PAHs in two environmental water samples, and a small amount of ANY (1.39 μg/L) was detected in one water sample. Satisfactory recoveries (82.5%-113.9%) were obtained when both water samples were spiked with the three PAHs at three levels. The experimental results indicate that the established analytical method can detect the three PAHs in environmental water samples.

多环芳烃(PAHs)是一类最早发现且数量最多的具有“致癌、致畸、致突变”性质的环境污染物,其污染面广,来源多,一直是环境领域重点关注的对象^[1,2]^。日常生活中,造成PAHs排放到大气、土壤、河流中的因素很多,例如化工厂废物排放、垃圾填埋不规范、烟熏油炸类食品制作不符合标准等^[3,4]^。这不仅对环境造成严重污染,通过食物链富集后,PAHs进入人体内,还会导致人体肺功能受损或肺癌的概率逐渐增大。因此,建立一种检测环境样品中PAHs含量的分析方法十分必要。然而,由于环境样品基质复杂且PAHs的含量较低,直接通过仪器进行检测,不仅减少仪器使用寿命,更会严重影响检测结果。因此在进行仪器分析之前,需要对环境样品进行必要的前处理。

目前有很多方法可用于PAHs样品的前处理,例如固相微萃取(SPME)技术、磁固相萃取(MSPE)技术、液相微萃取(LPME)技术等^[5⇓⇓⇓⇓-10]^。其中,SPME技术具有易于处理、溶剂消耗低、易于结合气相色谱等优势,成为一种应用广泛的样品前处理技术^[11⇓⇓-14]^。该技术的原理是目标分析物在涂层材料和样品溶液之间的分配平衡,因此萃取材料的特性对于萃取效果的灵敏度和选择性起着至关重要的作用^[12]^。目前市面上常见的涂层有聚二甲基硅氧烷(PDMS)、聚二甲基硅氧烷-二乙烯基苯(PDMS-DVB)、聚乙二醇-二乙烯基苯(PEG-DVB)等,由于它们在机械强度、稳定性、成本等方面尚存在不足,不能满足所有样品的检测^[13]^。为了提高萃取性能,许多新型材料被研制出并用作SPME涂层,例如:聚离子液体涂层、碳基纳米材料涂层、金属有机骨架复合材料涂层、分子印迹材料涂层等^[15⇓⇓⇓⇓⇓⇓-22]^。其中,导电聚合物是应用较广的涂层材料之一。在导电聚合物中,聚吡咯(Ppy)由于易于制备、成本低、绿色环保等优势常被用于提取极性芳香族化合物^[23]^,但将单一的聚吡咯用作SPME涂层制备的材料具有热稳定性较差、萃取效率较低等缺陷。聚吲哚(PIn)及其衍生物富含大量的*π-π*共轭体系,可与极性目标分析物之间产生强烈的相互作用,在SPME领域也有较大的应用潜力,将其与导电性能良好的吡咯(py)共聚,可以得到具有较高萃取效率的SPME涂层。目前尚未见将吡咯与2,3,3-三甲基吲哚(2,3,3-TMe@In)共聚制备SPME涂层的报道。氮化硼(BN)是一种具有较高热稳定性和比表面积的新型无机材料,常被用于药物传送和催化剂负载,在吸附污染物方面也得到了广泛的应用。例如,Pang等^[24]^合成了纤维状氮化硼纳米片,并采用磁固相微萃取的方式对10种农药进行吸附;Marchesini等^[25]^制备了六方氮化硼纳米片作为萃取涂层从水样中吸附多氯联苯。由此可见,BN在SPME领域也具有较大潜力。

基于此,本研究通过电化学循环伏安法制备了一种新型多孔氮化硼掺杂聚吡咯-2,3,3-三甲基吲哚(Ppy/P2,3,3-TMe@In/BN)复合材料,并将其作为SPME涂层,用于环境水样中3种PAHs的检测。通过扫描电子显微镜(SEM)、红外光谱(FTIR)、热稳定性分析进行表征,表征结果证明所制备的复合材料具有较高的热稳定性,该材料多孔多褶皱的结构,赋予其更大的比表面积,从而实现对PAHs的分离富集。将其修饰在不锈钢丝表面制成SPME涂层并与GC-FID相结合,建立了一种适用于检测环境水样中PAHs的分析方法。

## 1 实验部分

### 1.1 仪器与试剂

GC 2010-Plus型气相色谱仪,配有氢火焰离子化检测器(FID)和GC-solution色谱工作站(日本岛津仪器有限公司);自制固相微萃取手柄;CHI电化学工作站(辰华仪器有限公司,上海)用于固相微萃取涂层的电化学制备。三电极体系:不锈钢丝(2 cm×250 μm O. D.)为工作电极,铂丝(2.5 cm×0.1 cm O. D)为辅助电极,饱和甘汞电极(SCE)为参比电极。Quanta-200扫描电子显微镜(荷兰FEI公司); Nexus-670红外光谱仪(美国Nicolet公司)。用于比较的商用萃取头为表面涂有PDMS的熔融石英纤维(膜厚:65 μm,购于美国Supelco公司)。

吡咯(分析纯)、2,3,3-三甲基吲哚(分析纯)、十二烷基硫酸钠(SDS,纯度98%)均购于阿拉丁化学有限公司(上海),其中吡咯使用前需经过减压蒸馏进行纯化。氯化钠、三聚氰胺、尿素、硼酸、萘(naphthalene, NAP,分析纯)、苊(acenaphthylene, ANY,分析纯)、芴(fluorene, FLU,分析纯)均购自上海国药集团化学试剂公司。

### 1.2 色谱条件

HP-5毛细管柱(30 m×0.25 mm×0.25 μm,兰州中科安泰分析科技有限责任公司);色谱柱室的程序升温设定:50 ℃保持3 min,以19 ℃/min的速率升温至200 ℃,不停留;以4.5 ℃/min的速率升温至250 ℃,维持3 min;再以2 ℃/min的速率升温至260 ℃,维持3 min。采用不分流进样模式。气化室和检测器温度均设置为280 ℃。超纯N_2_作为载气,流速为1 mL/min。

### 1.3 标准溶液配制

质量浓度为10 mg/L的多环芳烃标准溶液用甲醇配制,并于4 ℃冰箱内保存,后续按照实验要求稀释成所需浓度的系列PAHs标准工作溶液。

### 1.4 Ppy/P2,3,3-TMe@In/BN复合SPME涂层的制备

#### 1.4.1 多孔BN的制备

采用三聚氰胺、硼酸以及尿素经过高温碳化制备BN。具体实验步骤如下:在研钵中加入三聚氰胺(1.02 g)、尿素(2.40 g)和硼酸(0.50 g)并混合均匀。随后,把混合物放入石英舟内,并在管式炉内通N_2_ 0.5 h,在10 ℃/min的升温速率条件下加热至800 ℃,并维持210 min。降温后,得到白色BN固体。

#### 1.4.2 Ppy/P2,3,3-TMe@In/BN复合涂层的制备

将2 cm长的不锈钢丝分别用1 mol/L HNO_3_、1 mol/L NaOH和去离子水超声清洗15 min,以除去表面杂质。随后,将三电极插入含有25 mg SDS、90 μL py、30 μL 2,3,3-TMe@In和8 mg BN的10 mL水溶液中。扫描电位范围为-0.2~1.6 V,扫描圈数为25圈,扫描速度为80 mV/s。制备完成后,将涂层清洗干净,在60 ℃下的烘箱中干燥24 h再进行老化处理。管式炉程序升温设置条件如下:100 ℃老化30 min, 250 ℃老化2 h。最后将老化后的萃取头粘在自制的SPME手柄上。在相同条件下,制备Ppy、Ppy/Pin、Ppy/P2,3,3-TMe@In和Ppy/BN涂层。

### 1.5 样品预处理

首先通过自然沉降除去环境水样中的泥沙及大颗粒杂质,其次采用抽滤法除去样品中存在的植物纤维等细小杂质,最后利用0.45 μm的水系滤膜过滤2次,得到样品溶液。

### 1.6 SPME萃取过程

取10 mL 300 g/L的氯化钠水溶液于萃取瓶中,随后加入15 μg/L的 NAP、ANY、FLU混合标准溶液和磁子,用橡胶塞密封好后,放于恒温磁力搅拌器上。当升温至45 ℃时,将萃取涂层暴露于萃取瓶中的顶空部位,在600 r/min下对3种PAHs进行顶空-固相微萃取(HS-SPME)。萃取35 min后,将萃取头快速收回于保护针筒内,并迅速插进气相色谱进样口,在280 ℃条件下解吸5 min,用色谱峰面积评价复合SPME涂层的性能和萃取效率。

## 2 结果与讨论

### 2.1 制备条件考察

#### 2.1.1 py和2,3,3-TMe@In体积比的优化

聚合单体的体积比对复合涂层的厚度有一定的影响,厚度过薄会导致涂层不稳定,厚度过厚易使涂层上的孔隙被封堵,从而降低萃取效率。因此,本研究优化了py和2,3,3-TMe@In的体积比。通过改变py和2,3,3-TMe@In的体积比(即1∶5、1∶3、1∶1、3∶1、5∶1、7∶1)制备出相应的涂层。研究发现,当py含量较少且吲哚(In)含量较多时,得到的涂层非常稀薄,分布不均匀,且容易脱落,这表明In的导电性较差,使得共聚物不能稳定地修饰在不锈钢丝表面。随着py含量的不断增加,得到的涂层慢慢变得致密均匀。如图1a所示,当py和2,3,3-TMe@In的体积比为3∶1时,对PAHs的萃取效率最高,因此,在后续研究中采用体积分别为90 μL和30 μL的py和2,3,3-TMe@In制备复合涂层。

**图 1:**
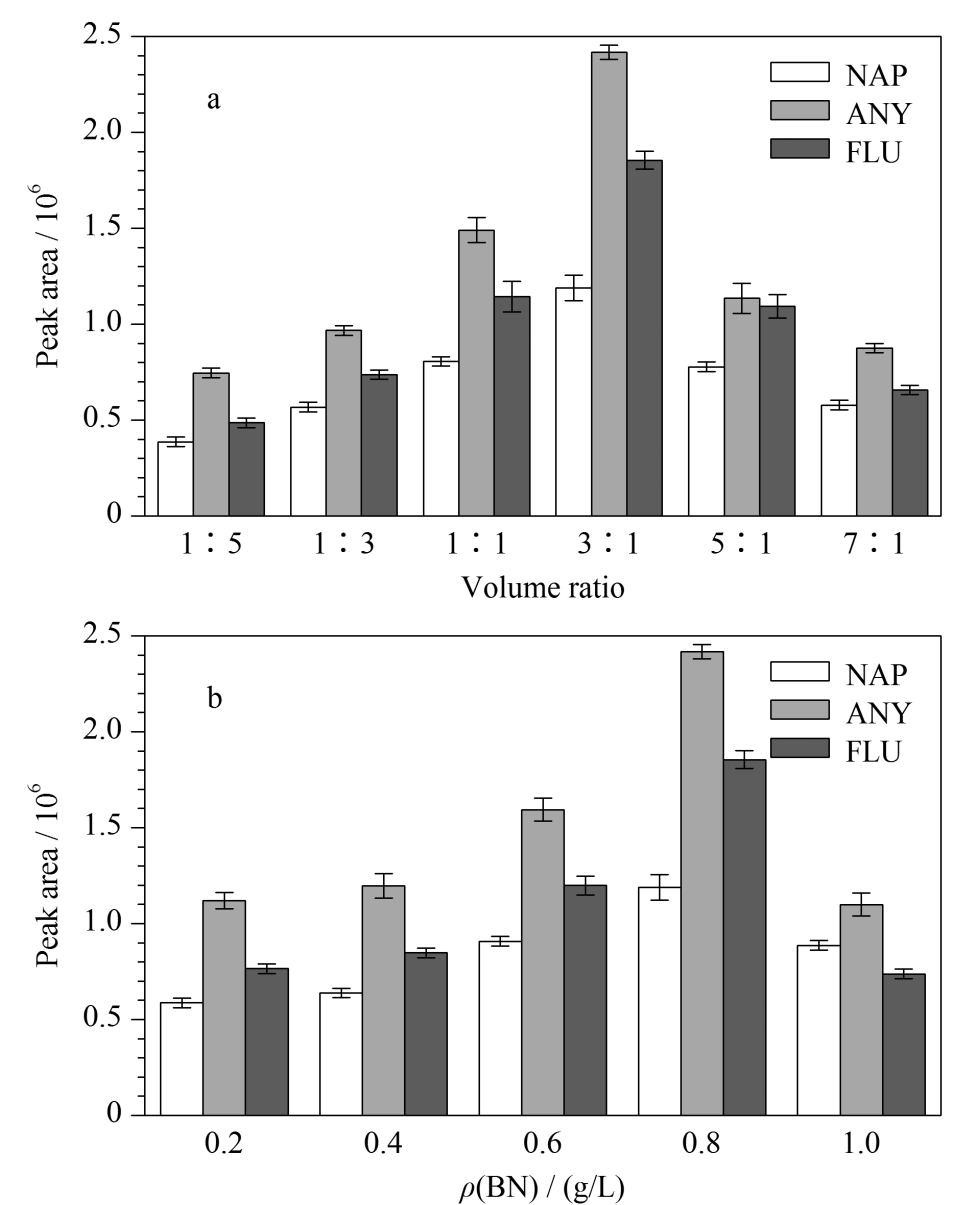
(a)py与2,3,3-TMe@In的体积比与(b)BN质量浓度对Ppy/P2,3,3-TMe@In/BN萃取多环芳烃效率的影响(*n*=3)

#### 2.1.2 BN浓度的优化

BN的加入,可以提高复合SPME涂层的热稳定性。为了获取最佳BN浓度,研究了质量浓度分别为0.2、0.4、0.6、0.8、1.0 g/L的BN对萃取效率的影响。如图1b所示,随着BN浓度的增加,萃取效率不断增加;这是因为BN的加入增大了复合材料的比表面积。但当BN浓度大于0.8 g/L时,萃取效率反而下降。主要原因是较高浓度的BN会在溶液中团聚,使得制备的涂层比表面积减小。在后续研究中将0.8 g/L作为BN的最佳浓度。

**图 2:**
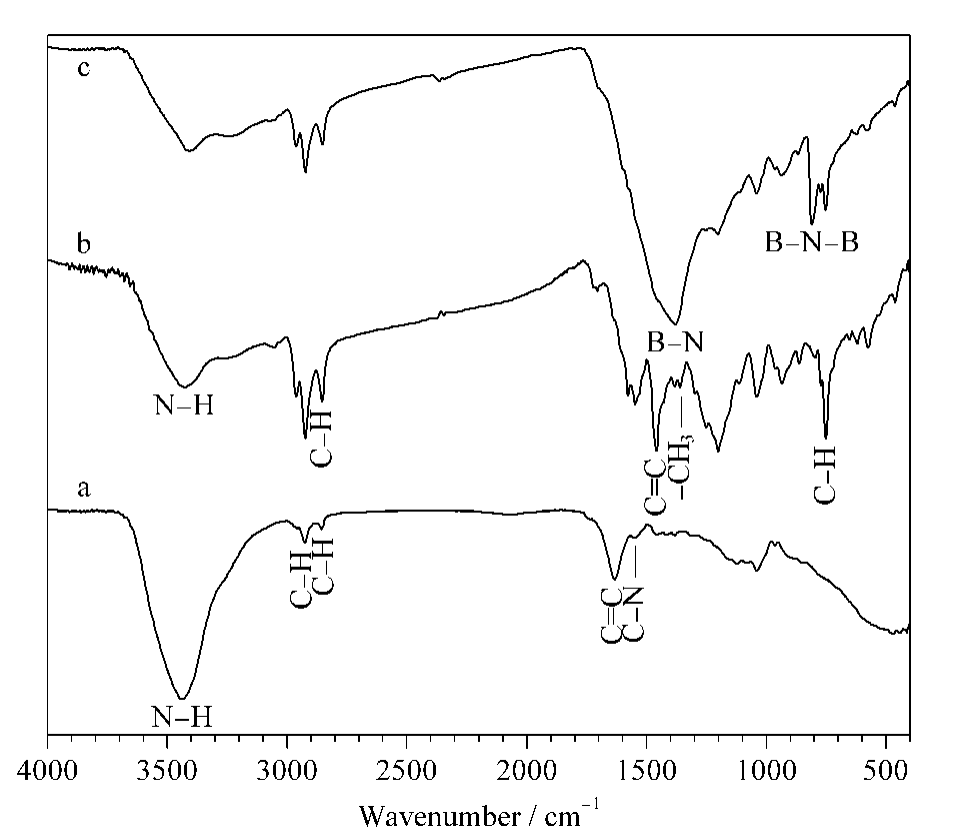
(a) Ppy、(b) Ppy/P2,3,3-TMe@In和(c) Ppy/ P2,3,3-TMe@In/BN的红外光谱图

### 2.2 Ppy/P2,3,3-TMe@In/BN复合涂层的表征

#### 2.2.1 红外表征

图2是Ppy、Ppy/P2,3,3-TMe@In和Ppy/P2,3,3-TMe@In/BN的FTIR图。对比Ppy和Ppy/P2,3,3-TMe@In图可知,Ppy在3400 cm^-1^处的峰是Ppy中N-H的振动峰;1650 cm^-1^处的峰是Ppy中C=C的振动峰;2800和3000 cm^-1^处的峰归属于Ppy中C-H的伸缩和变形振动峰。在Ppy/P2,3,3-TMe@In曲线中,可以看出在1380 cm^-1^处小而尖的峰属于P2,3,3-TMe@In中-CH_3_的振动峰,P2,3,3-TMe@In中的C-H振动峰位于650 cm^-1^处。从曲线c可以看出,位于1385和809 cm^-1^处的峰是BN中B-N的伸缩和变形振动峰(与文献^[25]^中BN的特征峰一致)。通过上述分析可知,Ppy和P2,3,3-TMe@In双单体共聚且有BN掺杂的复合涂层成功制备。

#### 2.2.2 热稳定性分析

通过将萃取头放入色谱进样口采集基线的方式,考察了Ppy/P2,3,3-TMe@In/BN复合涂层的热稳定性。如图3所示,Ppy/P2,3,3-TMe@In/BN在320 ℃的高温下基线仍然较为平整。当温度高达340 ℃时,Ppy/P2,3,3-TMe@In/BN的基线变得不稳定,表明Ppy/P2,3,3-TMe@In/BN复合涂层有分解物出现。由此可知,该涂层具有良好的热稳定性。

**图 3:**
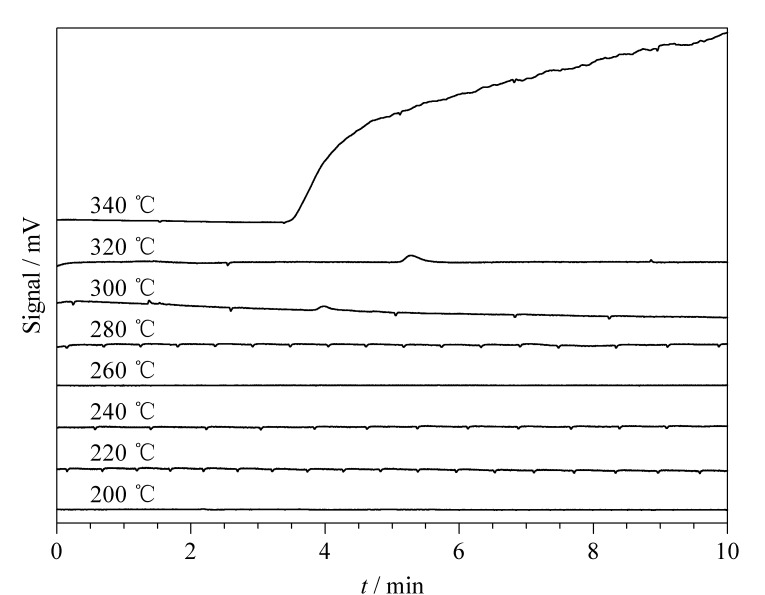
Ppy/P2,3,3-TMe@In/BN涂层的热稳定性

#### 2.2.3 表面结构表征

图4分别是BN、Ppy、Ppy/P2,3,3-TMe@In和Ppy/P2,3,3-TMe@In/BN涂层的SEM图。从图4a可以看出BN呈现出多孔结构;纯的Ppy是表面类似于花椰菜的结构(图4b);从图4c中可以看出具有树枝状结构的In镶嵌在Ppy中。图4d是复合涂层的萃取头扫描电镜图,该涂层呈现出多孔多褶皱的枝状结构,即Ppy/P2,3,3-TMe@In/BN复合涂层制备成功,该结构有利于增强涂层对PAHs的吸附能力。

**图 4:**
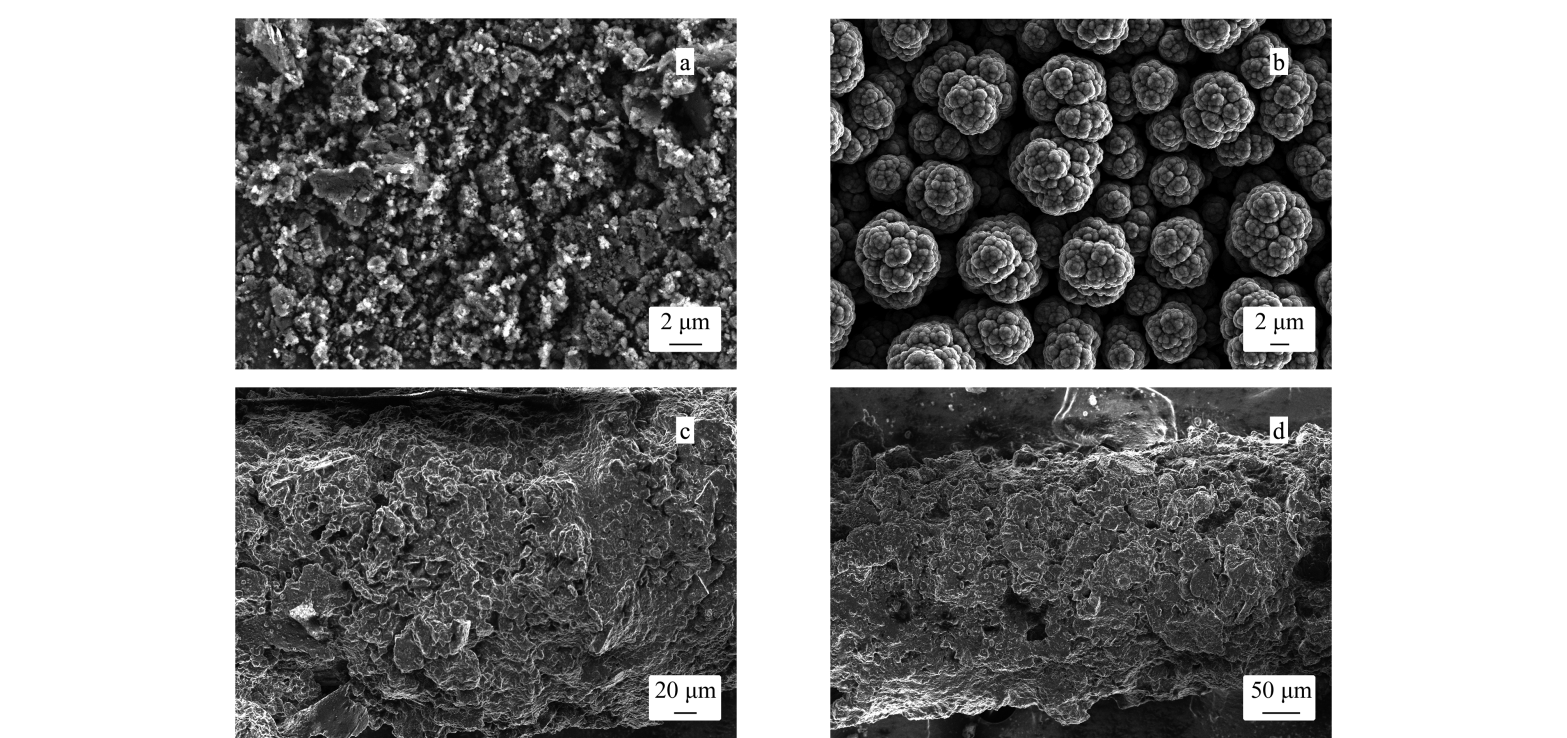
(a)BN、(b)Ppy、(c)Ppy/P2,3,3-TMe@In和(d)Ppy/P2,3,3-TMe@In/BN涂层的SEM图像

### 2.3 HS-SPME条件优化

#### 2.3.1 萃取条件优化

萃取效率受萃取条件的制约。本工作采用单因素分析法考察了不同萃取温度、萃取时间、盐溶液浓度、搅拌速度对萃取效率的影响。萃取过程中采用的PAHs标准溶液的质量浓度均为15 μg/L,每一组实验重复3次。

首先,考察了萃取温度在25~65 ℃时,复合SPME涂层萃取效率的变化。如图5a所示,在一定范围内,萃取温度越高,萃取效率越大,这是由于温度升高加快了分子热运动。当萃取温度为45 ℃时,萃取效率最大。由于萃取吸附过程是放热过程,且温度升高萃取基质中的少量水分会附着于涂层表面,因此继续升高温度,萃取效率变小。在后续的实验中,将萃取温度设置为45 ℃。

**图 5:**
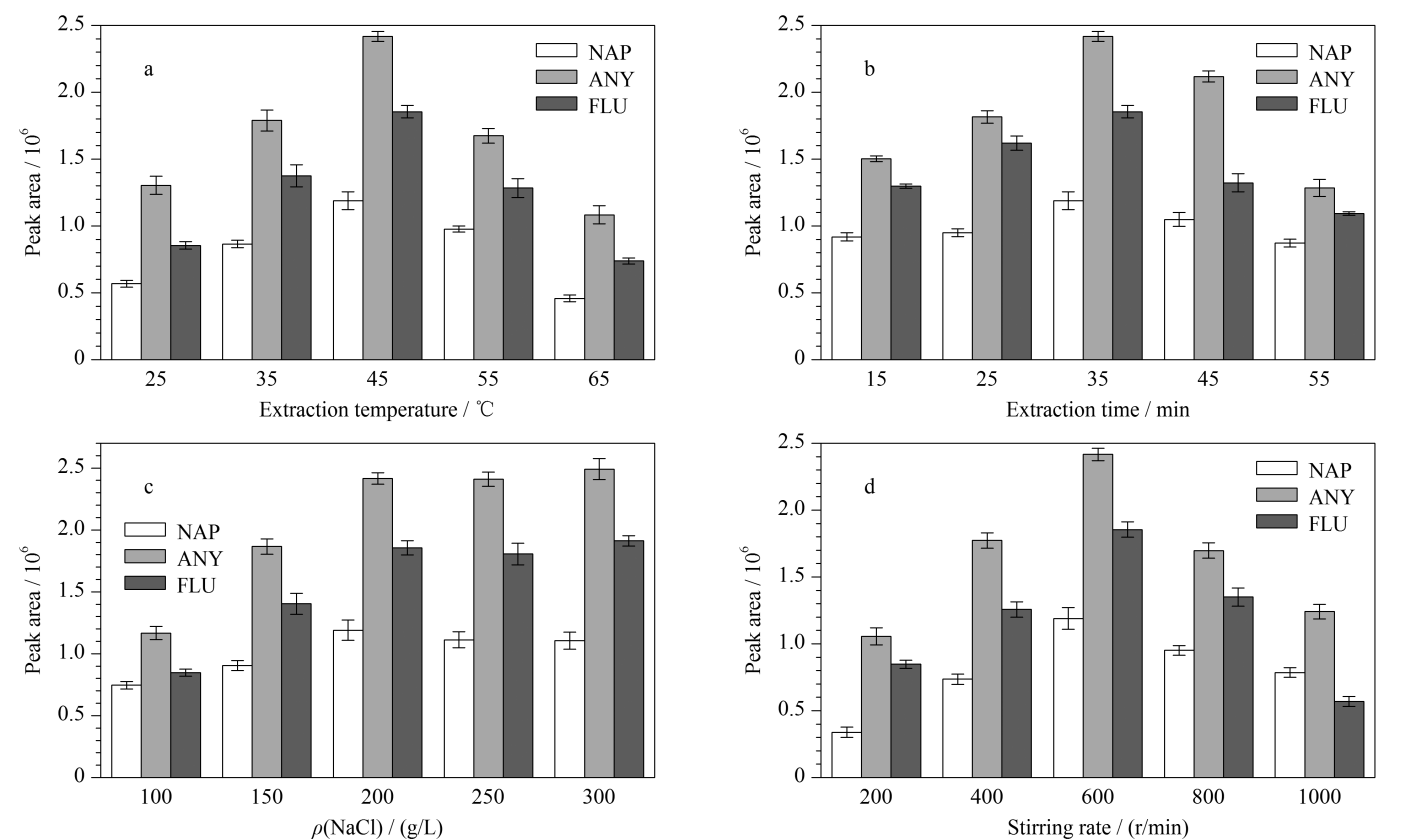
(a)萃取温度、(b)萃取时间、(c)NaCl质量浓度和(d)搅拌速率对Ppy/P2,3,3-TMe@In/BN涂层萃取效率的影响(*n*=3)

随后,进一步考察了萃取时间在15~55 min范围内,复合SPME涂层萃取效率的变化。一般来说,在萃取达到平衡之前,萃取时间越长,萃取效率越大。但随着萃取时间的推移,竞争吸附也会有一定程度的增加。如图5b所示,当萃取时间为35 min时,复合涂层对PAHs的萃取效率均较好。因此,在后续的实验中,将35 min作为最佳萃取时间。

为了提高SPME的萃取效率,一般采用氯化钠盐析法。因此,考察了质量浓度范围为100~300 g/L时氯化钠基质溶液对萃取效率的影响。如图5c所示,随着氯化钠浓度的增加,萃取效率相应地有所增加。当盐浓度到达300 g/L(接近饱和状态)时,萃取效率达到最大,这可能是由于盐浓度的提高,增加了样品溶液在顶空中的蒸汽压,有利于目标分析物向顶空分配。因此在后续实验中,将质量浓度为300 g/L的氯化钠溶液作为工作溶液。

同时,搅拌速度会影响目标分析物在水相和顶空相之间的传质。在一定范围内,搅拌速度越大,萃取效率越高。如图5d所示,当转速在200~600 r/min范围内时,萃取效率随着搅拌速度的增加而增加。当转速大于600 r/min时,复合SPME涂层的萃取效率逐渐下降,这是由于磁力搅拌器中的搅拌子不稳定,导致少量水汽附着在涂层表面从而影响萃取效率。因此,选择转速为600 r/min。

#### 2.3.2 解吸条件优化

在解吸过程中,影响萃取效率最直接的因素是解吸温度和解吸时间。为得到最佳的萃取效率,考察了解吸温度(220~300 ℃)和解吸时间(1~9 min)对复合SPME涂层萃取效率的影响。

由图6a可知,随着解吸温度的升高,萃取效率逐渐增加,当温度到达280 ℃时,萃取效率最高,再升高温度不仅易导致目标分析物分解,还会影响涂层的稳定性。因此,将280 ℃作为最佳解吸温度。由图6b可知,随着解吸时间的增加,解吸过程逐渐平衡。当时间为5 min时,萃取效率达到最大。因此,在后续实验中,将解吸时间设置为5 min。

**图 6:**
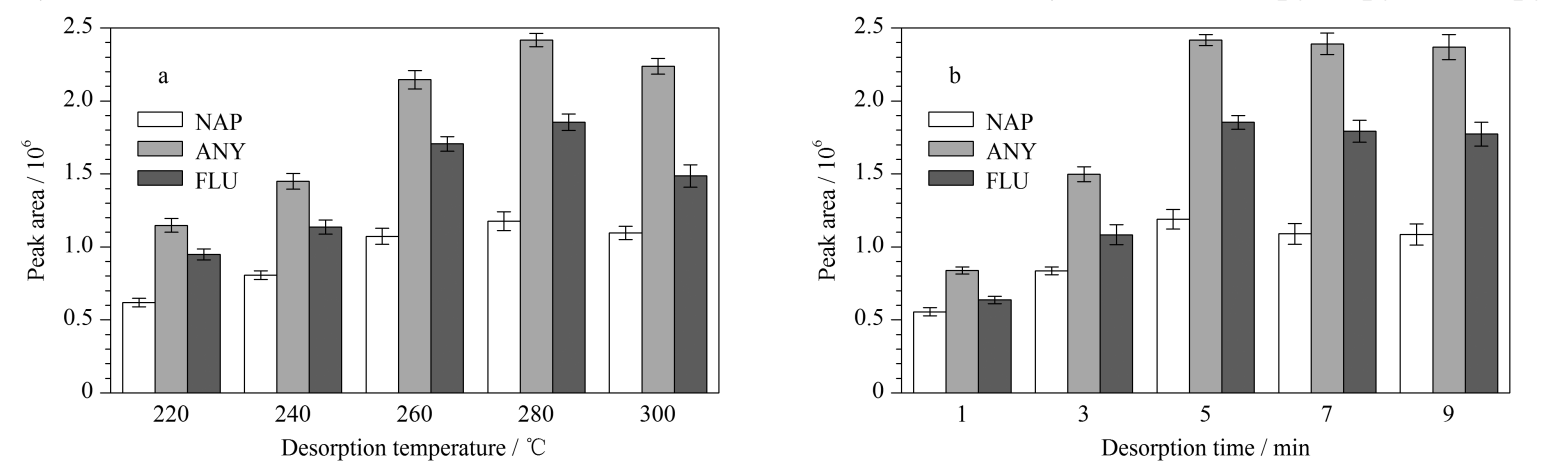
(a)解吸温度和(b)解吸时间对目标物质解吸效果的影响(*n*=3)

通过考察SPME过程中的萃取和解吸条件,获得了最佳实验条件,即:萃取温度为45 ℃,萃取时间为35 min,盐溶液质量浓度为300 g/L,搅拌速率为600 r/min,解吸温度为280 ℃,解吸时间为5 min。

### 2.4 方法评价

#### 2.4.1 萃取效率对比

目前,常见的商用SPME涂层有SiO_2_、PDMS等。本研究将所制备的SPME涂层与商用涂层PDMS作对比,通过制备Ppy、Ppy/Pin、Ppy/P2,3,3-TMe@In、Ppy/BN、Ppy/P2,3,3-TMe@In/BN SPME涂层,并在最佳萃取和解吸条件下,与商用涂层PDMS(厚度为100 μm)的萃取效率进行对比。如图7所示,它们的萃取效率从大到小为Ppy/P2,3,3-TMe@In/BN>Ppy/P2,3,3-TMe@In>PDMS>Ppy/BN>Ppy/PIn>Ppy。这表明Ppy/P2,3,3-TMe@In/BN涂层与PAHs之间的吸附作用力更强,具有较高的萃取效率。

**图 7:**
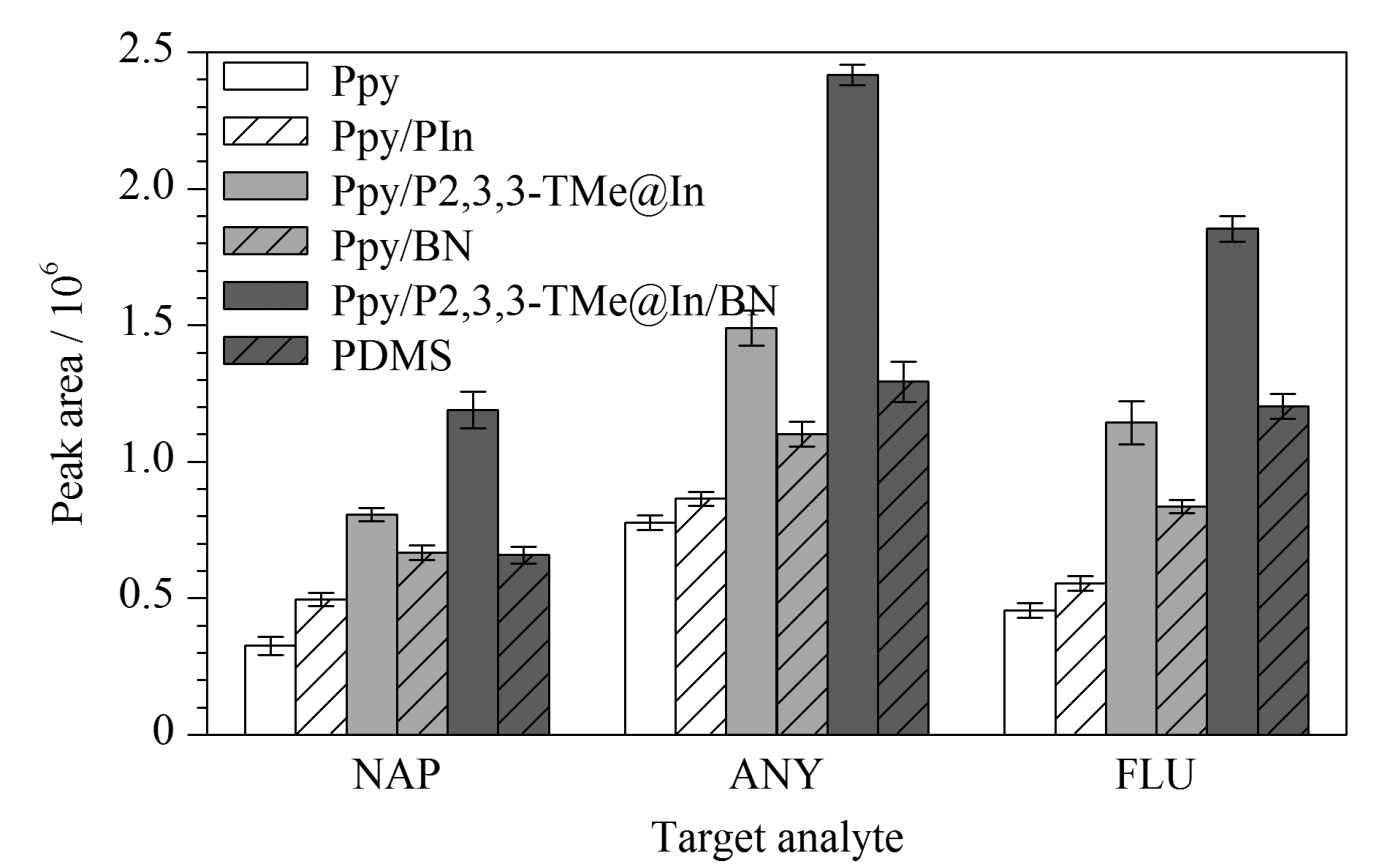
Ppy、Ppy/PIn、Ppy/P2,3,3-TMe@In、Ppy/BN、Ppy/P2,3,3-TMe@In/BN和PDMS对多环芳烃的萃取效率比较(*n*=3)

#### 2.4.2 检测体系的建立与验证

本研究分析了Ppy/P2,3,3-TMe@In/BN涂层的性能参数,结果见表1。该复合SPME涂层在所建立的SPME-GC-FID方法下,测定PAHs的线性范围为0.05~80 μg/L,相关系数为0.9968~0.9983,检出限(LOD)为10.6~14.5 ng/L(*S/N*=3)。对质量浓度为15 μg/L的PAHs工作溶液进行检测,一根萃取头连续测定5次的相对标准偏差(RSD)为5.41%~7.73%, 5根萃取头连续测定5次的RSD为9.13%~12.1%。由此可见,Ppy/P2,3,3-TMe@In/BN涂层具有较低的检出限及较好的重复性。

**表 1:** 基于Ppy/P2,3,3-TMe@In/BN涂层的SPME-GC-FID法测定多环芳烃的分析参数

Analyte	LOD/ (ng/L)	Linear range/ (μg/L)	Regression equation	Correlation coefficient	RSDs/%	
					One fiber (n=5)	Fiber to fiber (n=5)
NAP	10.6	0.05-80	Y=3.43×10^4^X+8.0×10^5^	0.9974	5.41	9.13
ANY	14.5	0.05-80	Y=9.20×10^4^X+1.4×10^6^	0.9968	7.73	12.1
FLU	12.8	0.05-80	Y=7.53×10^4^X+8.1×10^5^	0.9983	6.37	9.94

*Y*: peak area; *X*: mass concentration, μg/L.

此外,通过对该涂层的循环使用性进行考察,如图8所示,该萃取涂层在进行约120次吸附/解吸的情况下,萃取效率基本稳定。

**图 8:**
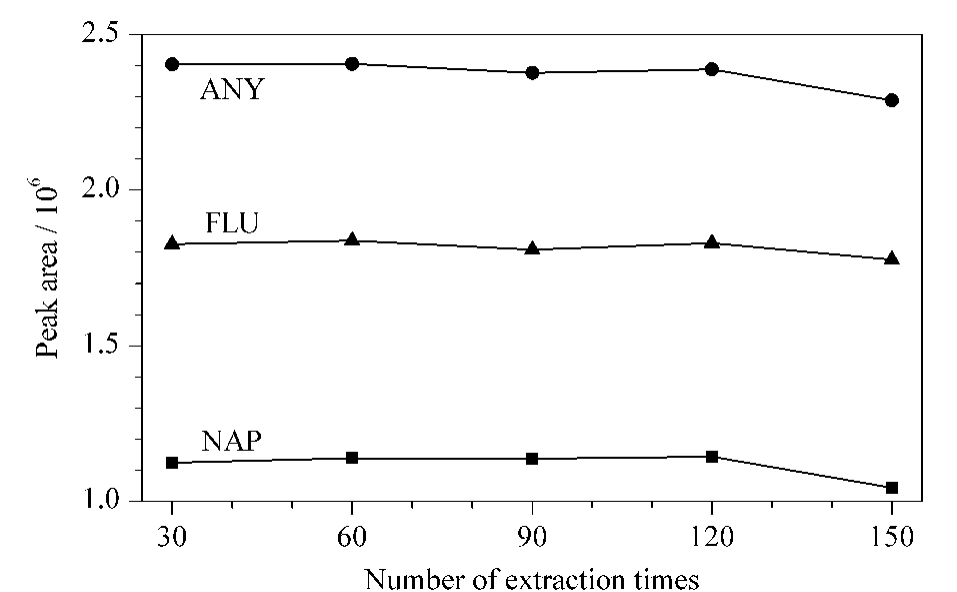
萃取效率随使用次数的变化

### 2.5 实际应用

将所建立的SPME-GC-FID分析方法用于环境水样中3种PAHs含量的检测。结果如表2所示,在水样1中检测到了少量的ANY,其含量为1.39 μg/L;水样2中未检测到PAHs的存在。为了进一步验证所建立的方法在实际样品中检测的可靠性,分别在水样1、2中加入低(1 μg/L)、中(10 μg/L)、高(50 μg/L)3个水平的标准溶液对本方法的准确度进行考察,所得出的加标回收率为82.5%~113.9%, RSD≤8.8%(*n*=3)。图9为水样1、2以及加标回收率试验的色谱图。实验结果表明,所建立的SPME-GC-FID方法具有良好的准确度,适用于环境水样中3种PAHs的分析。

**表 2:** 3种PAHs在环境水样中3个水平下的加标回收率及精密度(*n*=3)

Sample No.	Analyte	Background/ (μg/L)	1 μg/L			10 μg/L			50 μg/L	
			Rec./%	RSD/%		Rec./%	RSD/%		Rec./%	RSD/%
1	NAP	ND	85.7	8.2		84.5	7.9		94.0	3.7
	ANY	1.39	88.4	7.5		113.9	6.4		110.0	5.1
	FLU	ND	83.5	7.1		93.6	8.2		101.0	5.3
2	NAP	ND	87.4	6.7		86.1	5.0		96.3	4.9
	ANY	ND	92.8	8.8		100.6	6.9		105.0	2.8
	FLU	ND	82.5	6.9		108.7	8.3		102.0	3.6

Rec.: recovery; ND: not detected or lower than the LODs.

**图 9:**
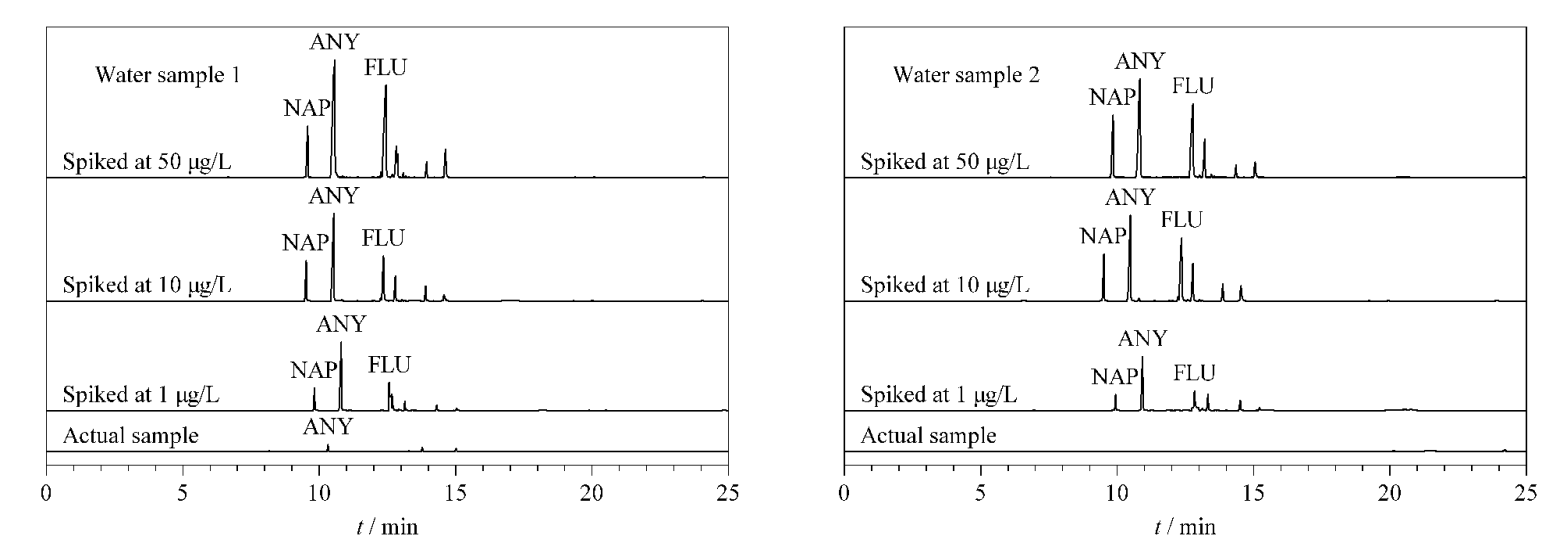
水样1和水样2的色谱图

### 2.6 与其他方法的对比

将所建立的分析方法与其他文献报道的方法进行比较(见表3)。本工作所建立的方法具有检出限低、回收率较高等优势。同时,该材料较好的热稳定性和循环使用性能为本方法的精密度和准确度提供了保障,可以满足水样中NAP、ANY、FLU 3种PAHs的同时检测。

**表 3:** 本方法与文献报道的水样中PAHs检测方法的比较

Coating	Method	Analytes	LODs/(ng/L)	Rec./%	RSDs/%	Ref.
ZIF-8	SPME-GC-FID	ANY, FLU	40-230	88.3-105.3	3.1-7.3	[26]
TiO_2_NTs/Ti	SPME-HPLC	NAP, FLU	30-50	97.8-111	3.7-8.9	[27]
Ppy/P2,3,3-TMe@In/BN	SPME-GC-FID	NAP, ANY, FLU	10.6-14.5	82.5-113.9	2.8-8.8	this work

ZIF-8: zeolite imidazole framework compound-8; TiO_2_ NTs/Ti: titanium dioxide nanotube fibers.

## 3 结论

本研究通过循环伏安法将py、2,3,3-TMe@In、BN制备成为Ppy/P2,3,3-TMe@In/BN复合SPME涂层,该涂层表现出良好的吸附性。将其与GC-FID结合并对萃取和解吸条件进行优化,建立了一种具有较低检出限、较高重复性的分析方法。该方法用于环境水样中NAP、ANY、FLU含量的检测,在水样1中检测到1.39 μg/L的ANY。相比于商用涂层,该涂层具有制备方法简单、富集效果好等优势,同时适用于复杂水样中3种PAHs的分离检测。
